# Development and evaluation of a simple PCR assay and nested PCR for rapid detection of clarithromycin-resistant *Helicobacter pylori* from culture and directly from the biopsy samples in India

**DOI:** 10.1186/s13099-023-00530-7

**Published:** 2023-02-14

**Authors:** Bipul Chandra Karmakar, Sangita Paul, Surajit Basak, Manisha Ghosh, Piyali Mukherjee, Rajashree Das, Sujit Chaudhuri, Shanta Dutta, Asish Kumar Mukhopadhyay

**Affiliations:** 1 Division of Bacteriology, ICMR-National Institute of Cholera and Enteric Diseases, Kolkata, 700010 India; 2 Division of Bioinformatics, ICMR-National Institute of Cholera and Enteric Diseases, Kolkata, 700010 India; 3 Amity Institute of Biotechnology, Amity University, Noida, Uttar Pradesh India; 4 AMRI Hospital Salt Lake, Kolkata, India

**Keywords:** *Helicobacter pylori*, Clarithromycin resistance, Antimicrobial resistance, Nested PCR

## Abstract

**Background:**

Eradication of *Helicobacter pylori* provides the most effective treatment for gastroduodenal diseases caused by *H. pylori* infection. Clarithromycin, a member of the macrolide family, still remains the most important antibiotic used in *H. pylori* eradication treatment. But the increasing prevalence of clarithromycin resistant *H. pylori* strains due to point mutations in the V region of the 23S rRNA, poses a great threat in treating the ailing patients. So, we aimed for PCR-mediated rapid detection of the point mutation at 2143 position of 23S rRNA gene in *H. pylori* that is relevant to clarithromycin resistance from culture and simultaneously from biopsy specimens to avoid the empirical treatment.

**Results:**

Newly developed PCR assay using DNA of pure culture detected point mutation in 23S rRNA gene in 21 (8.04%) of 261 clinical strains tested. The agar dilution method showed that all these 21 strains were resistant to clarithromycin indicating the perfect match of the PCR based results. Additionally, the sequencing study also identified the A to G mutation at 2143 position in 23S rRNA gene of the resistant strains only. Consequently, the newly developed Nested-ASP-PCR dealing directly with 50 biopsy specimens demonstrated 100% sensitivity and specificity with the findings of agar dilution method taken as Gold standard. Bioinformatics based analysis such as accessibility analysis and dot plot clearly stated that the base pairing probability has increased due to mutation. Computational studies revealed that the point mutation confers more stability in secondary structure due to conversion of loop to stem. Furthermore, interaction studies showed binding affinity of the CLR to the mutant type is weaker than that to the wild type.

**Conclusion:**

This assay outlines a rapid, sensitive and simple approach to identify point mutation that confers clarithromycin resistance as well as clarithromycin sensitive strains, providing rapid initiation of effective antibiotic treatment. Additionally, it is simple to adopt for hospital based diagnostic laboratories to evaluate the degree of regional clarithromycin resistance from biopsy specimens itself. Furthermore, in silico studies provide evidence or a signal that the prevalence of clarithromycin resistance may rise in the near future as a result of this point mutation.

## Introduction

Stomach cancer is the 5th most common neoplasm and the 3rd most deadly cancer, with an estimated 7,83,000 deaths in 2018 worldwide, remains as a serious threat in human population [1, 2] and importantly, *Helicobacter pylori* (*H. pylori*) infection is one of the robust known risk factors for gastric cancer. Most of the infections caused by the Gram-negative, spiral-shaped microaerophilic bacterium *H*. *pylori* are asymptomatic, 10–15% of them develop peptic ulcer disorders, stomach cancer or mucosa-associated-lymphoid-tissue (MALT) lymphoma [3–6]. Particularly in underdeveloped nations like India, *H. pylori* infection causes childhood malnutrition and, in fact, raises the risk of infection by other gastrointestinal infections such as *Vibrio cholerae* [7]. In India, *H. pylori* infections affect 60–70% of the population [8, 9]. So, if someone suffers from gastroduodenal diseases along with the detection of *H. pylori* infection, eradication of *H. pylori* provides the most effective treatment. Moreover, Gastric MALT-lymphoma became the first malignant tumor that has been reversed by antimicrobial therapy [10]. According to the recommendation of the National Institutes of Health Consensus Development Conference, first-line therapy to treat *H. pylori* infection associated with peptic ulcers is a combination of two antibiotics, clarithromycin (CLR), amoxicillin, or metronidazole, with proton pump inhibitors such as omeprazole or pantoprazole, prescribed without performing susceptibility testing. However, the efficiency of the conventionally used empirical treatment of triple therapy has been drastically reduced [11–13].

Emerging antibiotic resistance is the primary reason for the failure of *H. pylori* eradication therapy, however other concerns such as incorrect medication, poor patient compliance, and intermittent antibiotics use also need consideration [14]. In India, 90% *H. pylori* strains are resistant to metronidazole. However all *H. pylori* strains are found to be susceptible to amoxicillin [15]. It has been reported that the prevalence of resistance to CLR has increased significantly in many countries in recent years [16–21]. A recent retrospective observational study in a large cohort (4744 *H. pylori*-infected individuals) from Hungary found that the prevalence of CLR resistance was 17.2% overall during the examined period (2005–2013) [22]. One Japanese study concluded that the prevalence of CLR resistance had significantly raised from 1.8% in 1996 to 27.1% in 2008 [23], and another group reported that patients examined between 2000 and 2013 in the Japanese population had an overall resistance rate of 31.1% [24]. Similarly, from 14.8% in 2000 to 52.6% in 2014, China has encountered a surge in CLR resistance [25, 26]. Additionally, Korea experienced a sharp rise in the prevalence of CLR resistance, jumping from 11% in 2005 to 60% in 2009 [27, 28]. In 2005 a study conducted from Kolkata, India no strains were identified as being CLR-resistant. However, a study from 2016 found that 11.8% of the *H. pylori* isolates from North India were resistant to CLR [29, 30].

Hence, CLR resistant *H. pylori* positions as one of the most problematic bacterial infections and is enrolled in the World Health Organization (WHO) priority list of antibiotic-resistant bacteria [31] which drives expeditious study of this bacterium.

Many studies have proved that structural change of the 23S rRNA is the main reason for showing bacterial resistance towards this antibiotic. These structural changes are caused due to single nucleotide polymorphism (SNP) of 23S rRNA [32]. According to research by Versalovic et al*.* the point mutation from A to G transition at positions 2143 and 2144 in the domain of the 23S rRNA which corresponds to 2058 and 2059 in *E. coli*, is primarily responsible for the development of CLR resistance [33].

Normally, phenotypic methods such as susceptibility testing which includes agar dilution method or E-test are employed for assessment of CLR resistance. Although, these tests lack sensitivity and take around 1 week for informative results [29]. Moreover, the mentioned phenotypic methods lack of providing any genetic mutational information regarding bacterial resistance. To detect mutation, many molecular-based methods such as PCR/DNA-enzyme immunoassay [34], PCR-based denaturing HPLC assay [14], PCR restriction fragment length polymorphism (RFLP), nested PCR [35–37] and most recently ARMS-PCR [38] have been employed. Although these tests demonstrated a high level of sensitivity and specificity, their effectiveness is insufficient to deliver quick laboratory results, and they are occasionally difficult to execute.

In order to resolve these multifaceted technical constraints and to comprehend the CLR susceptibility pattern of our concerned strains, the present study is conducted about the development of Mismatch Amplification Mutational Assay (MAMA) PCR for quick identification of the A to G point mutation at 2143 position in V domain of 23S rRNA genes of *H. pylori* that is relevant to CLR resistance from culture and concurrent establishment of nested-allele specific primer-polymerase chain reaction (nested-ASP-PCR) directly from biopsy specimens to discriminate the resistant allele from sensitive one and to get the overall scenario regarding antibiotic resistance towards CLR as well in India.

In addition, the secondary structure of 23S rRNA was investigated based on the accessibility profile and change of base pairing probability of mutant type in reference to the wild type strain. Then, the impact of mutation (A2143G) on 23S rRNA secondary structure and natural selection (if any) on the evolution of 23S rRNA structural element at 2143 position were also examined. Moreover, on the basis of their tertiary structures, the interaction between CLR with wild-type and with mutant 23S rRNA were examined to understand the potential of a strain towards drug susceptibility.

## Materials and methods

### Biopsy sample collection

A total of 261 *H. pylori* strains were used in this study, including 158 strains that were archived between the years of 2005 and 2015 and 103 strains that were isolated from 180 adult subjects of both sexes (aged between 21 and 71 years) with gastroduodenal abnormalities who underwent endoscopy at hospitals in various parts of India during the years of 2016 and 2018. Complete socio-demographic information and health records were collected from well-informed and consenting patients using a standardized questionnaire. After detailed medical assessment, endoscopy was performed on such patients according to the procedures as mentioned in the institutional ethical clearance. Each patient’s whole medical history was concealed during the research procedure, and during the data analysis, the disease status was uncovered. During endoscopy, two biopsy samples were obtained from each patient: one from the antrum and the other from the stomach corpus. The biopsy samples were transferred to the Bacteriology Division of the National Institute of Cholera and Enteric Diseases and stored at − 70 °C until further processing in 0.8 ml of Brucella broth containing 15% glycerol (Difco Laboratories, Detroit, MI).

### Phenotypic assay

#### *Helicobacter pylori* culture

In laboratory, the transport media with the biopsy specimens were vortexed for 15 min and 100 µl of the media along with the specimen was spread on Blood agar plates containing brain–heart infusion agar (BHIA) (Difco Laboratories) supplemented with 7% sheep blood, 0.4% IsoVitaleX (Becton–Dickinson, San Jose, CA, USA), polymyxin B (8 µg/ml), trimethoprim (5 µg/ml), vancomycin (6 µg/ml) and nalidixic acid (8 µg/ml) (all from Sigma Chemicals).The plates were incubated for 3-4 days at 37°C in a double gas incubator (Heraeus Instruments) in an atmosphere of 5% O_2_, 10% CO_2_ and 85% N_2_. Gram staining, rapid urease, catalase, and oxidase test findings, as well as genotyping results from the urease PCR, were used to identify and confirm *H. pylori* colonies based on their translucent water droplet appearance [39]. The *H. pylori* isolates were preserved in BHI broth with 20% glycerol at – 80 °C. DNA isolated from CLR sensitive *H. pylori* strain, 26695 and CLR resistant *H. pylori* strain, 197 from Delhi [30] were taken as control for standardization of PCR.

#### Phenotypic antimicrobial susceptibility test by agar dilution method

To assess the sensitivity and specificity of this novel protocol, the observations were compared to the phenotypic identification of the sensitive and resistant strains by agar dilution method which is the “Gold standard” method. A stock solution of CLR (1 mg/ml) (Sigma) was prepared in acetone and then filter sterilized. 24-h old *H. pylori* culture was suspended in phosphate buffer saline (PBS) and absorbance adjusted to McFarland standard 0.5 ~ approximately 10^8^ CFU/ml. Then tenfold serial dilution of this suspension was prepared and 10 µl from each serially diluted bacterial suspension was applied on BHI agar media with the appropriate concentrations of CLR (0.125, 0.25, 0.5, 1.0 or 2.0 µg/ml) and BHI agar media containing no antibiotic considered as growth control. Solvent control was taken to know the effect of the solvent on *H. pylori* growth if any. In this susceptibility testing, one reference strain (ATCC 43504) was considered as the control. The spots were dried, then incubated at 37 °C double gas incubator for 3-4 days to observe the growth. MIC break-point was considered according to the then EUCAST guideline, 2016.

### Extraction of genomic DNA from culture

Genomic DNA was extracted by the Phenol–chloroform extraction method. Briefly, a half loopful of 24 h grown healthy bacterial culture was harvested in 200 µl of Tris–EDTA buffer (pH 8.0) followed by rigorous vortexing and then centrifuged at 12,000 rpm for 10 min with a combination of phenol: chloroform: isoamyl alcohol (25:24:1) saturated with 10 mM Tris and 1 mM EDTA (Sigma-Aldrich, St Louis, MO, USA). The supernatant was once extracted using 150 µl of the mixture of chloroform: isoamyl alcohol (24:1) followed by centrifugation for 10 min at 12,000 rpm. And finally, the acquired DNA-containing supernatant served as the template for PCR investigation.

### PCR assay

A novel MAMA PCR has been designed by exploiting the single base mutation using 4 µl of diluted template (1:30). Three MAMA-PCR primers were developed, with one forward primer (2143F) being employed for both the sensitive as well as the resistant alleles of 23S rRNA and two allele-specific reverse primers, uniquely complementary to either sensitive or resistant type of 23S rRNA alleles (2143AR and 2143GR respectively). These two allele-specific reverse primers, each were designed with specific nucleotide at their respective 3ʹ ends; C for 2143GR (complementary to resistant type allele) and T for 2143AR (complementary to sensitive type allele). Furthermore, the 3ʹ mismatch effect was enhanced by introducing another alteration in the nitrogenous base in the second nucleotide position from the 3ʹ end of both the reverse primers (G in place of T)**.** Two separate PCR reactions were performed in a 20 µl reaction volume, one is for the detection of CLR sensitive strains and the other is for CLR-resistant strains, both containing genomic DNA (2–20 ng), 1.5 mM MgCl_2_ (Takara), 200 μM forward and reverse primers, 200 µM each dNTPs (Takara), 1 U Taq DNA polymerase (Takara), in a regular PCR buffer for 25 cycles, using the following reaction parameters: 95^○^C for 90 s, 95^○^C for 25 s, 60^○^C for 10 s, and 72^○^C 20 s, 72^○^C for 5 min and 4 °C for ∞ in a Master Cycler apparatus ( Eppendorf, Hamburg, Germany). 26695 was taken as a positive control for CLR sensitive PCR and 197 as a positive control for CLR resistant PCR respectively. Both types of PCR yielded an amplicon of 183 bp. The PCR results were examined using 2% agarose gels in 1X TAE buffer that contained 0.5 g of ethidium bromide per ml. With the use of Quantity One software, bands were observed under UV light (Bio-Rad, Hercules, CA). Molecular weight markers were used to establish the product’s size.

### Nucleotide sequencing of *H. pylori* 23S rRNA

Finally, to confirm the PCR-based identification of the point mutation responsible for the CLR resistance, randomly selected 13 representative strains were sequenced for the 23S rRNA. Primer sets (Table 1) 23S rRNA F and 23S rRNA R were used to amplify the 617 bp segment of the gene covering the peptidyltransferase region of 23S rRNA which contains point mutation responsible for CLR resistance [40]. A PCR amplification with volume of 50 µl reaction mixture containing 50–80 ng of genomic DNA, 200 µM of each primer, 1.5 mM Mgcl_2_ (Takara)_,_ 200 µM each dNTPs (Takara), and 2U of Taq (Takara) polymerase in a standard 10X buffer for 30 cycles with a following conditions: 96 °C for 2 min, 94 °C for 30 s, 60 °C for 30 s, and 72 °C 40 s, 72 °C for 5 min and 4 °C for ∞ in a Master Cycler apparatus was performed. Amplified PCR products were then purified by using Thermo scientific PCR purification kit. Cycle sequencing was carried out using the PCR purified product as template and purified again. Then the products from cycle sequencing reactions were loaded onto sample tubes, sealed with septa, and arranged on a 48-well tray. The tray was loaded to an automated sequencer (ABI PRISM 3100 Genetic Analyzer, Applied Biosystems). The raw sequencing data were analyzed using the ABI PRISM DNA Sequencing Analysis Software (Applied Biosystems).

**Table 1:** Primers used in this study for PCR amplification

Primers	Sequence (5ʹ–3ʹ)	Amplicon (bp)	References
2143F	GTA AAC GGC GGC CGT AAC TAT	183	This study
2143AR	GTA AAG GTC CAC GGG GTC GT		This study
2143GR	GTA AAG GTC CAC GGG GTC GC		This study
UreBF	CGT CCG GCA ATA GCT GCC ATA GT	480	[39]
UreBR	GTA GGT CCT GCT ACT GAA GCC TTA		[39]
23S rRNA F	GGC TCT TTG AGT CCT TTT AGG ACA A	617	[40]
23S rRNA R	CTC CAT AAG AGC CAA AGC CCT TAC T		[40]

### GenBank accession numbers of the gene sequences

In this work, the sequence discovered were deposited in GenBank under accession number (GenBank accession number MW080351 to MW080363).

### Isolation of DNA from biopsy samples

Template DNA was prepared directly from the biopsy sample according to Chattopadhyay et al*.* with slight modification [41]. Briefly, the biopsy specimen was vortexed for 5 min and 100 µl of supernatant without specimen was taken in 1.5 ml micro centrifuge tube. Then the tubes were boiled in a water bath for 15 min, immediately snap-chilled in ice and then centrifuged at 12000 rpm for 5 min. Now the supernatant was used as template.

### Nested PCR

#### Step 1

Amplification of 23S rRNA was performed using 3 µl template isolated from biopsy samples according to the protocol discussed by Wang et al*.* [40]. Briefly, primer sets (Table 1) 23S rRNA F and 23S rRNA R were used to amplify the 617 bp segment of the gene covering the peptidyltransferase region of 23S rRNA which contains point mutation responsible for CLR resistance. A PCR amplification with the volume of 20 µl reaction mixture containing a boiled template, 200 µM of each primers, 1.5 mM MgCl_2_ (Takara)_,_ 200 µM each dNTPs (Takara), and 2U (Takara) of Taq polymerase in a standard 10X buffer for 30 cycles with a following conditions: 96 °C for 2 min, 94 °C for 30 s, 60 °C for 30 s, and 72 °C 40 s, 72 °C for 5 min and 4 °C for ∞ in a Master Cycler apparatus was performed.

#### Step 2

Nested-ASP-PCR was performed utilizing 2 µl diluted PCR product (1:500) of the first round PCR following the same protocol as mentioned above in the PCR assay section.

To validate this observation, the nested PCR was performed in a conventional way, i.e. using the bacterial genomic DNA as the template. For this purpose, the *H. pylori* colonies were cultured on standard BHIA plates revived from the biopsy tissues and the genomic DNA was isolated from that culture.

### Computational prediction of rRNA secondary structures

Secondary structures of the two 23S rRNA transcripts were generated following the method proposed by Mathews et al*.* [42]. The minimum free energy for the secondary structure of single sequences using the dynamic programming was predicted by the proposed algorithm. The Z-score was calculated using the following formula:

1 $$\mathrm{Z}=\frac{<\mathrm{FFEpr}>}{\mathrm{STD}} - \frac{<\mathrm{FFEcr}>}{\mathrm{STD}}$$

Where, < FFEpr > is the average value of folding free energy of a partially randomized sequence and < FFEcr > is the average value of folding free energy of a completely randomized sequence. STD denotes the corresponding standard deviation. A negative Z-score indicates higher stability of a partially randomized sequence compared to a completely randomized sequence [43].

### Calculation of base pair probabilities

For the robust computation of the base pair probabilities of RNA sequences, the algorithm proposed by Bernhart et al. was used [44]. In this method, the frequency of a certain base pair (i, j) in local minimum energy structures that were computed using sequence windows of a given size L was considered. It derives recursions for the average equilibrium probability of a base pair (i, j) across all fixed-size sequence windows. Heat map matrices were used to depict the change in base pair probabilities of the 23S rRNA for wild type (A2143), mutant type (2143G), and their differences. For direct comparison, the wild type and mutant data were integrated into a single matrix. The base-pair probabilities were more clearly visualized by using colour codes. In a combined line plot, position-based accessibility data and its changes shown. An assessment of the effects of the mutation was done by the plot. It is beneficial to understand changes in highly accessible areas.

### Computational structure modelling and interaction study

The 3D structures of the 23S rRNA of wild type and mutant type *H. pylori* were created using the method proposed by Wang et al*.* [45]. The 3D structure of CLR was retrieved from DrugBank [46]. All the three structures in 3D format were refined in Discovery Studio visualizer v-2019 (discover.3ds.com). A molecular interaction study was carried out between CLR with both rRNAs using the method proposed by Honorato et al*.* with the default parameters [47].

### Statistical analysis

The sensitivity and specificity of the designed PCR assay was calibrated by a conventional 2X2 contingency table. The column showed the phenotypic resistance data i.e., “Gold standard” against CLR (resistant on left) whereas the PCR assay results were mentioned in the respective rows (resistant on top). True positive (left upper cell) represents positive PCR results confirmed by the gold standard. Count of “False positive” samples i.e., resistant by PCR but phenotypically sensitive were shown in the right upper cell. Lower left cell was referred to for false negatives (phenotypically resistant and PCR confirmed to be negative). “True negative” was shown in the lower left cell which is both phenotypically and PCR sensitive. The sensitivity and specificity were expressed following the guidelines mentioned by Mukherjee et al*.* [48].

## Results

Pure culture of clinical *H. pylori* strains was isolated from 261 patients and confirmed using different phenotypic biochemical tests followed by urease PCR [39].

### Development of a PCR assay based on a single mutation

CLR, a macrolide antibiotic, binds to the peptidyl transferase region of 23S rRNA of *H. pylori* and inhibits protein synthesis resulting in a bacteriostatic effect [49]. Our study has identified the A2143G point mutation in the 23S rRNA, responsible for CLR resistance in *H. pylori* [30]. In this study, our first purpose was to establish a PCR based assay that can comprehensively discriminate *H. pylori* strains carrying 23S rRNA A2143G mutation in a simple and rapid way and later on it can be used for diagnostic purposes to understand the dissemination of the new allele in different parts of India.

Three separate primers, one common forward primer (2143F) for both sensitive and resistant type 23S rRNA and two allele-specific reverse primers, uniquely complementary to either sensitive or resistant type of 23S rRNA alleles (2143AR and 2143GR respectively), were designed. Each of these allele-specific reverse primers, was designed with a specific nucleotide at their respective 3ʹ ends; C for 2143 GR (complementary to resistant type allele) and T for 2143 AR (complementary to sensitive type allele). Furthermore, the 3ʹ mismatch effect was enhanced by introducing another alteration in the nitrogenous base in the second nucleotide position from the 3' end of both the reverse primers (G in place of T)**.**

**Fig. 1:**
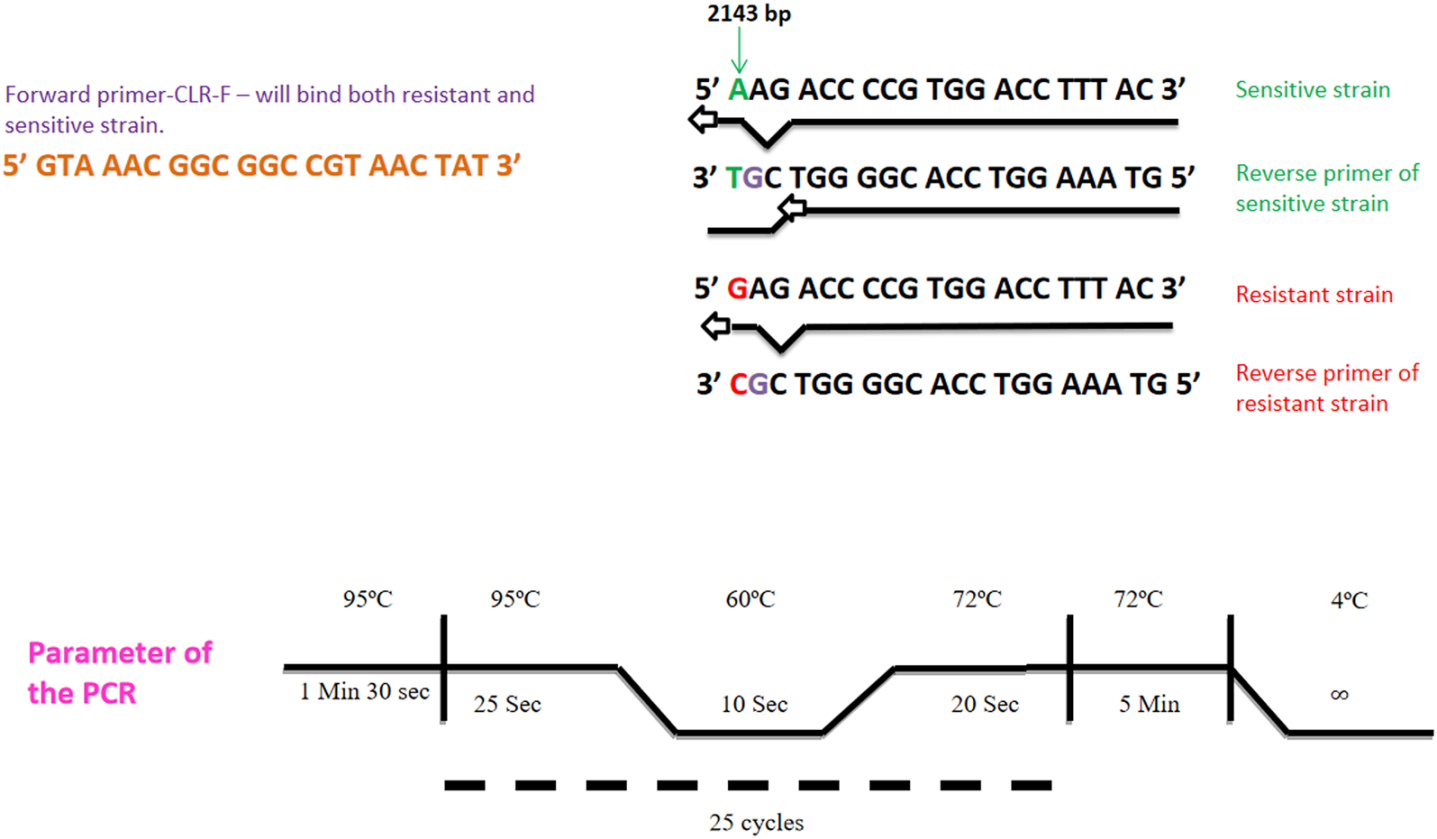
Design of new MAMA PCR to identify clarithromycin resistant strain: Three separate primers, one common forward primer 2143F marked as brick-red for both sensitive and resistant type 23S rRNA and two allele-specific reverse primers, were designed with specific nucleotide at their respective 3ʹ ends; C for 2143 GR (complementary to resistant type allele marked in red) and T for 2143 AR (complementary to sensitive type allele, marked in green). Additionally, the 3ʹ mismatch effect was enhanced by alteration of another nitrogenous base in the second nucleotide position from the 3ʹ end of both the reverse primers (G in place of T being marked as violet). Parameter of the PCR is described schematically the condition of PCR

We have standardized the PCR assay to optimize both the specificity and sensitivity using the protocol depicted in Fig. 1.

**Fig. 2:**
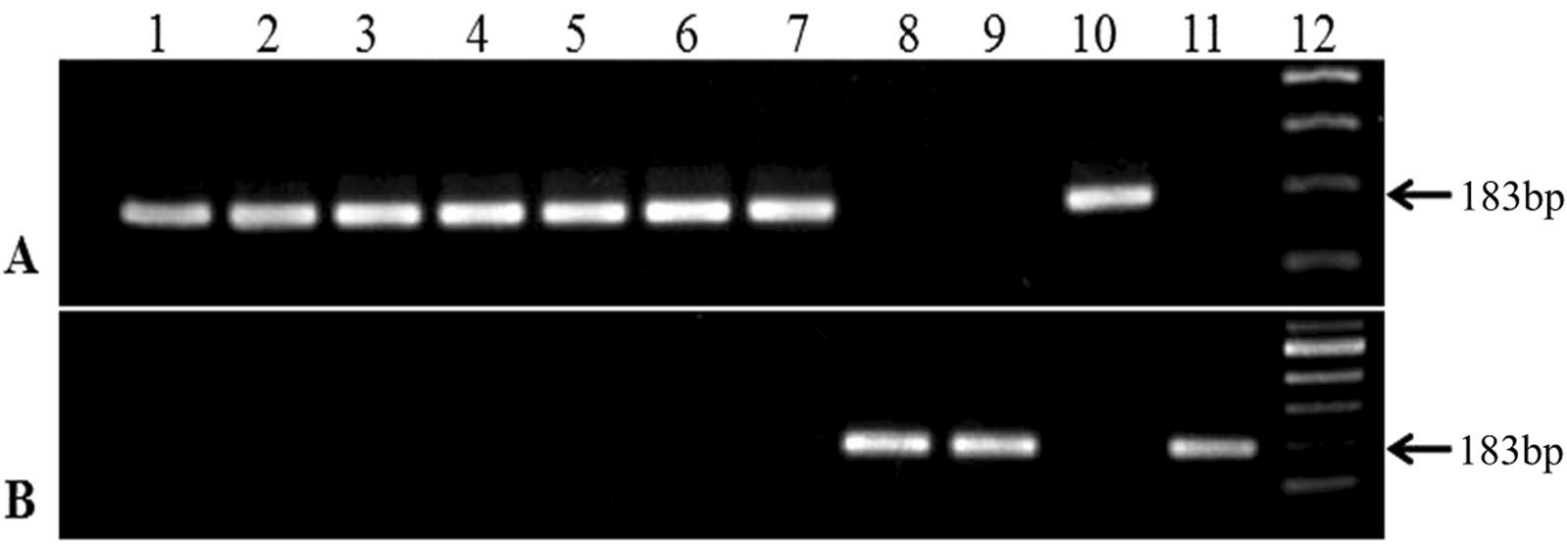
Development of PCR based assay for the detection of CLR sensitive and resistant type 23S rRNA of *H. pylori* isolates in India: MAMA-PCR to detect 23S rRNA in representative *H. pylor*i strains of India using primers (2143F/2143AR) for CLR-sensitive (**A**) and CLR-resistant (**B**). Lane1, Positive control for CLR-sensitive; Lane 2–10 represent clinical isolates; Lane 11, Positive control for CLR-resistant; Lane 12, DNA molecular size marker

Our newly designed PCR successfully differentiated the two different allelic subtypes of the 23S rRNA gene responsible for sensitive (wild type) and resistant (mutant) phenotype*. H. pylori* strains having CLR resistant allele yielded a 183-bp fragment of DNA with the primer pair 2143F and 2143GR but not with 2143F and 2143AR (Fig. 2). *H. pylori* strains having CLR sensitive allele depicted the reverse picture.

### Validation of the PCR assay

The evaluation of our newly designed PCR was monitored using template DNA from 261 clinical *H. pylori* isolates. Among the 261 *H. pylori* isolates tested, 21 isolates were produced amplicon for resistant allele (with primer set 2143F and 2143GR) but not for the sensitive allele specific primer set (2143F and 2143AR).Whereas, the remaining 240 strains yielded amplicon with the sensitive allele-specific primer set but not for the resistance alleles. On the other hand, susceptibilities of all the tested strains to CLR were determined by the agar dilution method and then compared with the PCR-based results. Among 261 isolates tested, 21 (8.04%) showed MIC > 0.5 µg/mL indicating that they are resistant to CLR. The remaining 240 strains showed varying level of susceptible MICs [MIC = 0.125 µg/mL (n = 196); MIC = 0.25 µg/mL (n = 35); and MIC = 0.5 µg/mL (n = 9)]. So, the result of the conventional antibiotic susceptibility assay perfectly matched with the PCR results. A conventional two-by-two (2X2) table was used to determine the accuracy of the PCR. A comparative account of the PCR-based technique and the conventional antimicrobial susceptibility assay i.e., gold standard was presented in Table 2. The sensitivity and specificity of the newly developed PCR assay was calculated according to Mukherjee et al*.* which showed 100% sensitivity and specificity, as both the techniques yielded same count of bacterial strains under sensitive and resistant group [48]. In addition, positive predictive and negative predictive values were also 100%.

**Table 2:** Specificity and sensitivity of the newly developed PCR assay using the conventional antibiotic susceptibility test

PCR assay	Conventional antibiotic susceptibility test		Total
	Resistant	Sensitive	
Resistant	21	0	21
Sensitive	0	240	240
Total	21	240	261

Sensitivity = 21/(0 + 21) × 100 = 100%

Specificity = 240/(0 + 240) × 100 = 100%

Positive predictive value = 21/(0 + 21) × 100 = 100%

Negative predictive value = 240/(0 + 236) × 100 = 100%

### Confirmation of the PCR-based results by sequencing analysis

To further confirm our PCR based result, 13 representative strains, which yielded amplicons with the primer pair 2143F and 2143GR specific for the resistant phenotype, were sequenced using two separate primers specific for the 23S rRNA gene producing amplicon of 617-bp. The results obtained from Nucleotide Sequence Analysis revealed that all the strains possessed DNA sequences similar to sensitive type of 23S rRNA (GenBank accession number U27270) except for a adenine to guanine substitution at the 2143 position (GenBank accession number MW080351 to MW080363) which was found to be associated with clarithromycin resistance of > 0.5µg/ml. Thus, the result of nucleotide sequencing confirms the results of newly designed PCR.

### Development of a nested-mismatch amplification mutation assay PCR to detect point mutation

**Fig. 3:**
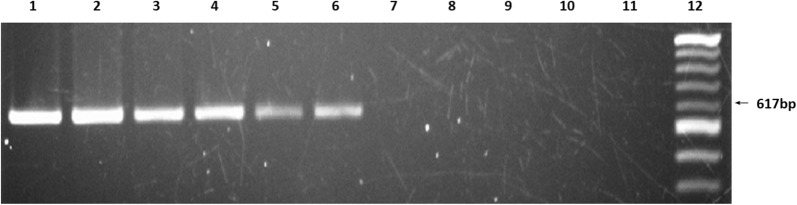
Amplification of 23S rRNA of *H. pylori* directly from gastric biopsy specimens: Identification of 23S rRNA of *H. pylori* by conventional single step PCR method. Lane 1 and 2 represent control CLR sensitive and resistant pure cultures respectively (DNA isolated by standard method), lane 3 represents control for CLR sensitive (DNA isolated by boiled template method), lane 4 to 11 represent gastric biopsy specimens (DNA isolated by boiled temple method) and Lane 12 represents 100 bp Ladder

The successful development and evaluation of the PCR assay for rapid detection of CLR- resistant *H. pylori* from the bacterial culture motivated us to develop a nested PCR assay for quick understanding of the presence of CLR-resistant *H. pylori* directly from the biopsy samples. It is a two-step PCR process. In the first step, 617-bp fragment of 23S rRNA gene containing the probable mutation site responsible for CLR resistance was amplified using the template DNA extracted from the biopsy samples. Few boiled template DNA from previously known culture positive biopsy samples and few culture negative samples were taken to standardize this PCR protocol. DNA of the standard strain, 26695 and CLR-resistant strain, 197 were used as positive controls to understand the successful completion of the first PCR. The findings from this PCR was quite interesting. The intensity of the band yielded from the amplification of genomic DNA isolated from pure culture following standard method [48] and that of band obtained from the amplification of boiled template DNA from pure culture were comparable and also intensity of both bands were more with respect to the band obtained from biopsy samples. But the perceptible fact was that some of the biopsy samples yielded bands where the others didn’t produce any band. Also the intensities were significantly different among PCR +ve biopsy samples. A possible explanation for this finding is that during *H. pylori* infection the bacteria colonizes in human gastric epithelium in patches and when these tissue samples are being collected via endoscopy it could be that the pinched-off tissue have various loads of bacterial population and thus yielding PCR amplified products of varying intensity. The boiled templates DNA from the pure culture and biopsy tissue samples which yielded a positive band had markedly varying intensity. Therefore it could be that, the biopsy tissue samples which apparently yielded no band in the first PCR, might have *H. pylori* infection with sub-optimal bacterial load yielding a PCR product that was not visible in gel electrophoresis (Fig. 3).

**Fig. 4:**
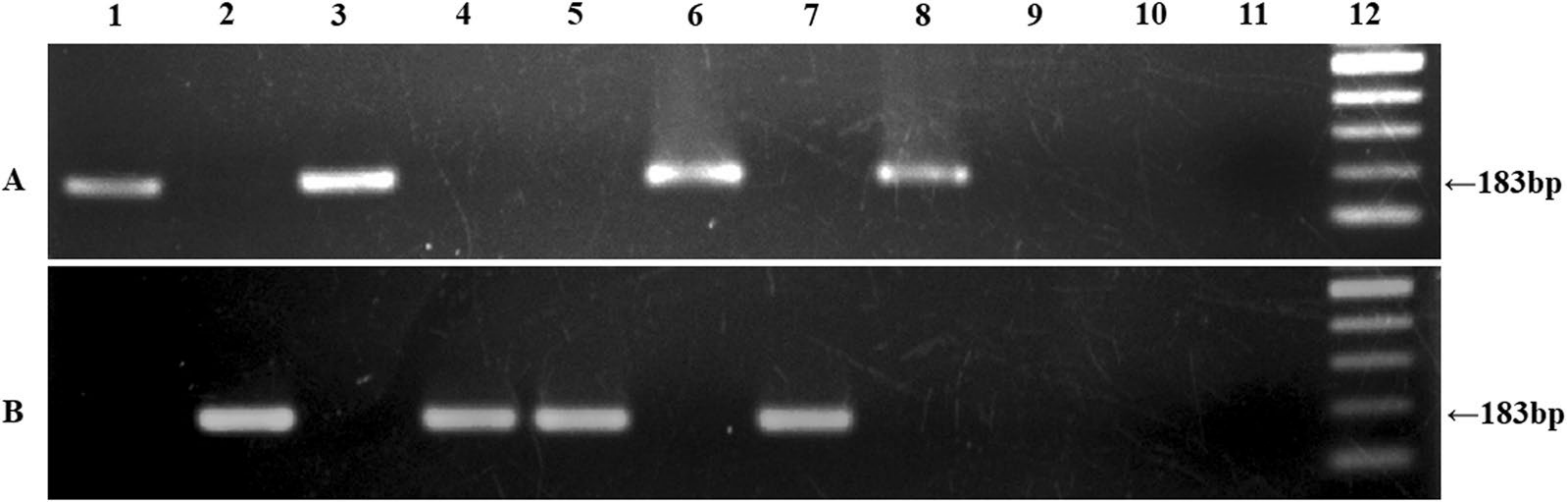
Detection of CLR sensitive and resistant type 23S rRNA of *H. pylori* isolates by nested-ASP-PCR: Identification and amplification of 23S rRNA sequence of *H. pylori* from human gastric biopsy specimens for CLR sensitive (**A**) and resistant type (**B**). Lane 1 and 2 represent control CLR sensitive and resistant pure cultures respectively (DNA isolated by standard method), Lane 3 represents control CLR sensitive (DNA isolated by boiled temple method), lane 4–11 represent gastric biopsy specimens (DNA isolated by boiled temple method) and Lane 12 represents 100 bp Ladder

To investigate this hypothesis, we performed nested PCR to enhance the sensitivity by running a second round of amplification with the previously amplified 617bp PCR product in the first PCR as template and allele-specific primers for CLR sensitive and resistant phenotype were used in this PCR to obtain 183-bp amplicons (Fig. 4). Stringent conditions were adapted to avoid false positive result as increased contamination is a common phenomenon in nested PCR. For the nested PCR, DNA from 50 biopsy samples were used. *H. pylori* isolated from 4 samples were CLR resistant and *H. pylori* from another 36 samples were CLR sensitive and the remaining 10 samples were *H. pylori* negative. The results of the nested PCR regarding CLR susceptibility perfectly matched the phenotypic gold standard data.

### Accessibility profiles of two alleles by computational analysis

**Fig. 5:**
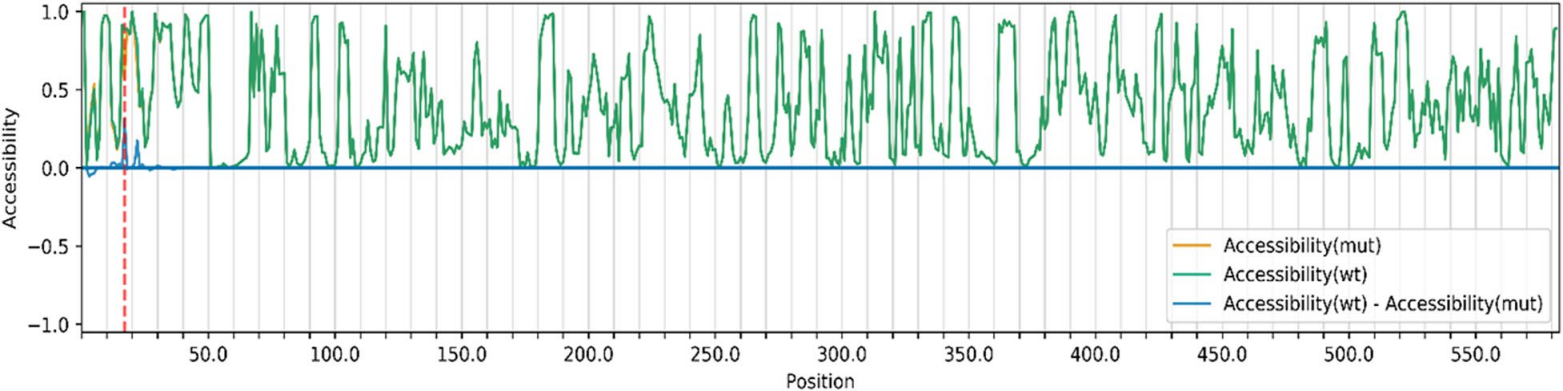
Accessibility profiles of wild type (green line) and mutant (yellow line) sequences and their differences (blue line): The mutated position is highlighted by a red line. The positive difference at the mutant position clearly indicates that wild type (A2143) has higher unpairing probability compared to mutant type (2143G)

The accessibility profiles of wild type (green line) and mutant (yellow line) sequences and their differences (blue line) provide an assessment of the mutation's effect on the RNA's single-strandedness, which is strongly related to its interaction potential with other RNAs. (Fig. 5). Accessibility is measured in terms of local single-position unpaired probabilities. The highlighted red line denotes the mutated position. The increased unpairing probability of the wild type (A2143) compared to the mutant type (2143G) is clearly shown by the positive difference (peak along the blue line) at the mutated location. This observation, in turn, suggests that the secondary structural stability of the wild type will be less stable compared to mutant type since the wild type has higher unpairing probability.

The impact of the mutation is also observed through change of base pairing probability induced by the mutation.

### Impact of the mutation by computational analysis

**Fig. 6:**
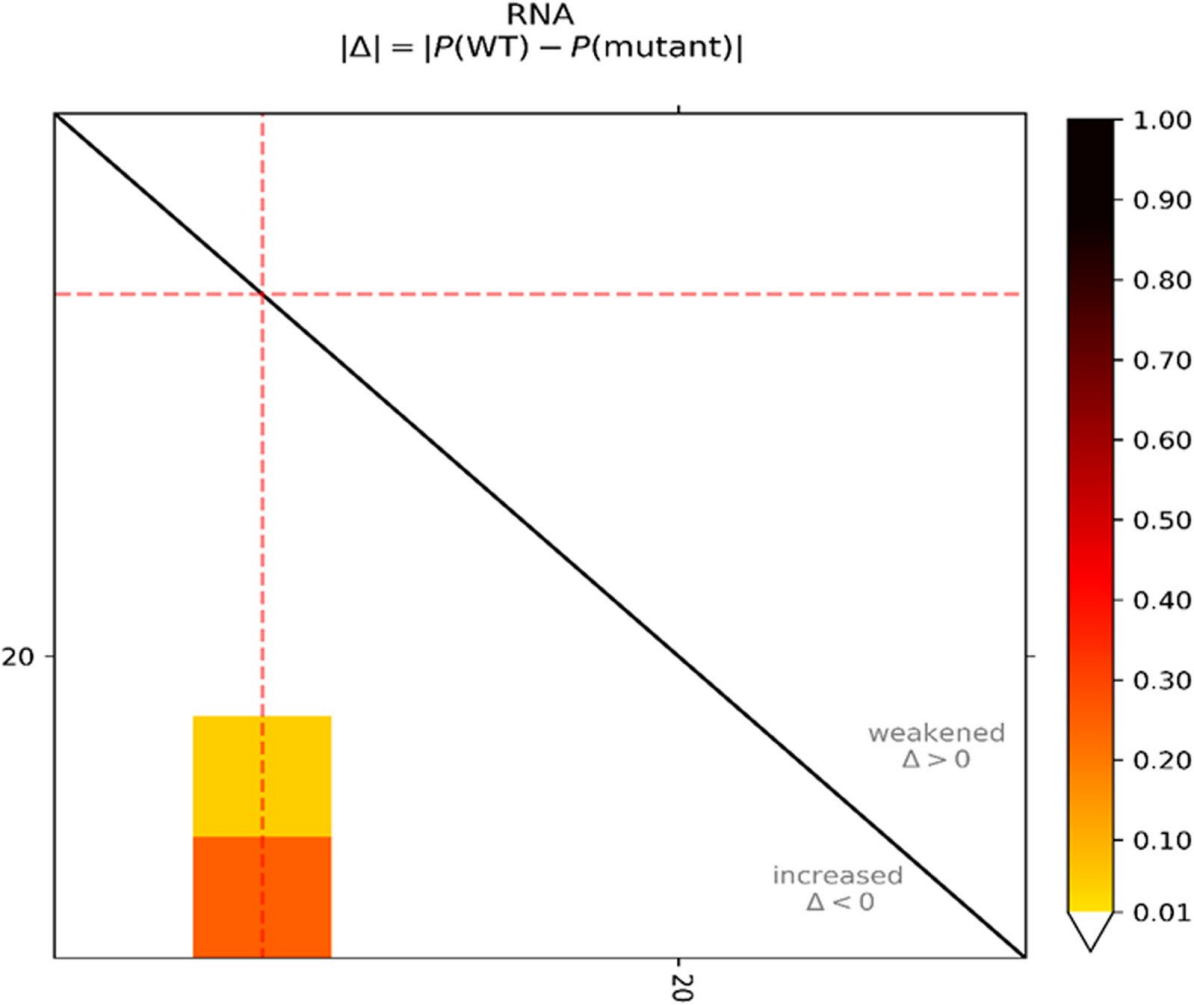
The dot plot (top-left) provides the absolute differences of the base pairing probabilities of wild type vs mutant 23S rRNA: The mutated position is highlighted by red dotted lines. Here, the grid box observed in the lower left part containing the mutated position confirms the increase of base pairing probability due to mutation

The absolute differences of the base pairing probabilities of wild type versus. mutant 23S rRNA, i.e. Pr(bp in wild type) - Pr(bp in mutant) is depicted in the top-left of the dot plot. The upper right part visualizes positive differences, i.e. a weakening of the base pairing potential, while the lower left reports negative differences, i.e. base pairs with increased probability within the mutant. Red dotted lines highlight the mutated position. The increase of base pairing probability due to mutation is confirmed by the grid box observed in the lower left part possessing the mutated position (Fig. 6). This observation also suggests that the secondary structural stability of the mutant form will be higher compared to wild type since the mutation has increased the base pairing probability.

The above two computational experiments undoubtedly suggest the need of 23S rRNA secondary structure analysis to ascertain impact of the mutation on the secondary structural stability.

### Impact of natural selection on the secondary structural stability of the two alleles

**Fig. 7:**
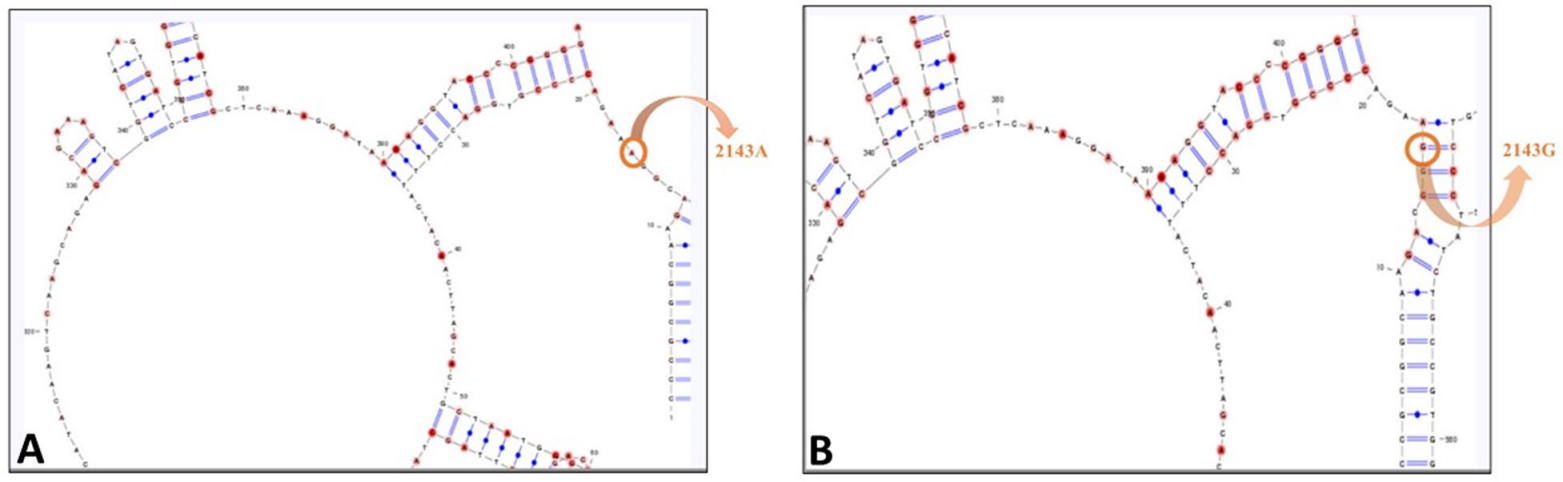
Predicted secondary structures of the 23S rRNA transcript in wild-type (2143A) and mutant form (2143G): **A** Secondary structure of rRNA transcript containing 2143A which is part of loop **B** Secondary structure of rRNA transcript containing 2143G which is part of stem structure. Conversion of loop into stem imparts higher stability of the 23S rRNA

The 23S rRNA secondary structures were designed for the two 23S rRNA sequences containing A2143 and 2143G. The two secondary structures are shown in Fig. 7 A, B. After comparison of the two figures, it is evident that the secondary structure has been changed at the mutational site. The mutant type, 2143G clearly forms a stem structure while the wild type, A2143 is part of a loop structure. We have compared the folding free energies of the two rRNA transcripts. The difference in folding free energy of rRNA transcript containing of the two variants is 3Kcal/mol with 2143G having more negative folding free energy. Lowering of folding free energy of rRNA transcript containing 2143G corresponds to a more stable secondary structure. Therefore, the conversion of loop into stem imparts higher stability of the rRNA transcript containing 2143G.

Many studies providing evidence for selection on RNA structure have employed a randomization protocol that shuffles nucleotide bases to generate numerous simulants. We randomized the rRNA transcript to generate 200 random sequences and measured the folding free energies for each random sequence. The average folding free energy for the complete set of 200 random sequences were calculated. We again performed the randomization of the rRNA transcript keeping a window of 20 nucleotides containing the A2143 mutation intact. Here also 200 random sequences were generated; the average folding free energy for the complete set of 200 random sequences were calculated and therefore the z-score was calculated (Z_A_). The same procedure was repeated for the rRNA transcript containing 2143G mutation and z-score was calculated (Z_G_). The value Z_A_ is − 1.384 Kcal/mol and the value of Z_G_ is − 2.916 Kcal/mol. A negative Z_A_ score indicates that the completely randomized rRNA sequence is less stable than the random sequence generated by preserving the A2143 in its original location. It indicates that natural selection has shaped the evolution of the rRNA sequence in such a way so that the preservation of residues around the position 2143 may impart higher stability to the secondary structure. As Z_G_ < Z_A_, the selection for the secondary structural stability of the rRNA sequence even becomes more when G is present at the 2143 position. Hence the stretch of sequence containing the position 2143 is important for imparting secondary structural stability of the rRNA. The variation of secondary structural stability of the two alleles would lead us to think about the potential reactivity/interactivity of the two alleles towards CLR.

### Drug sensitivity of the two alleles through computational interaction study

**Fig. 8:**
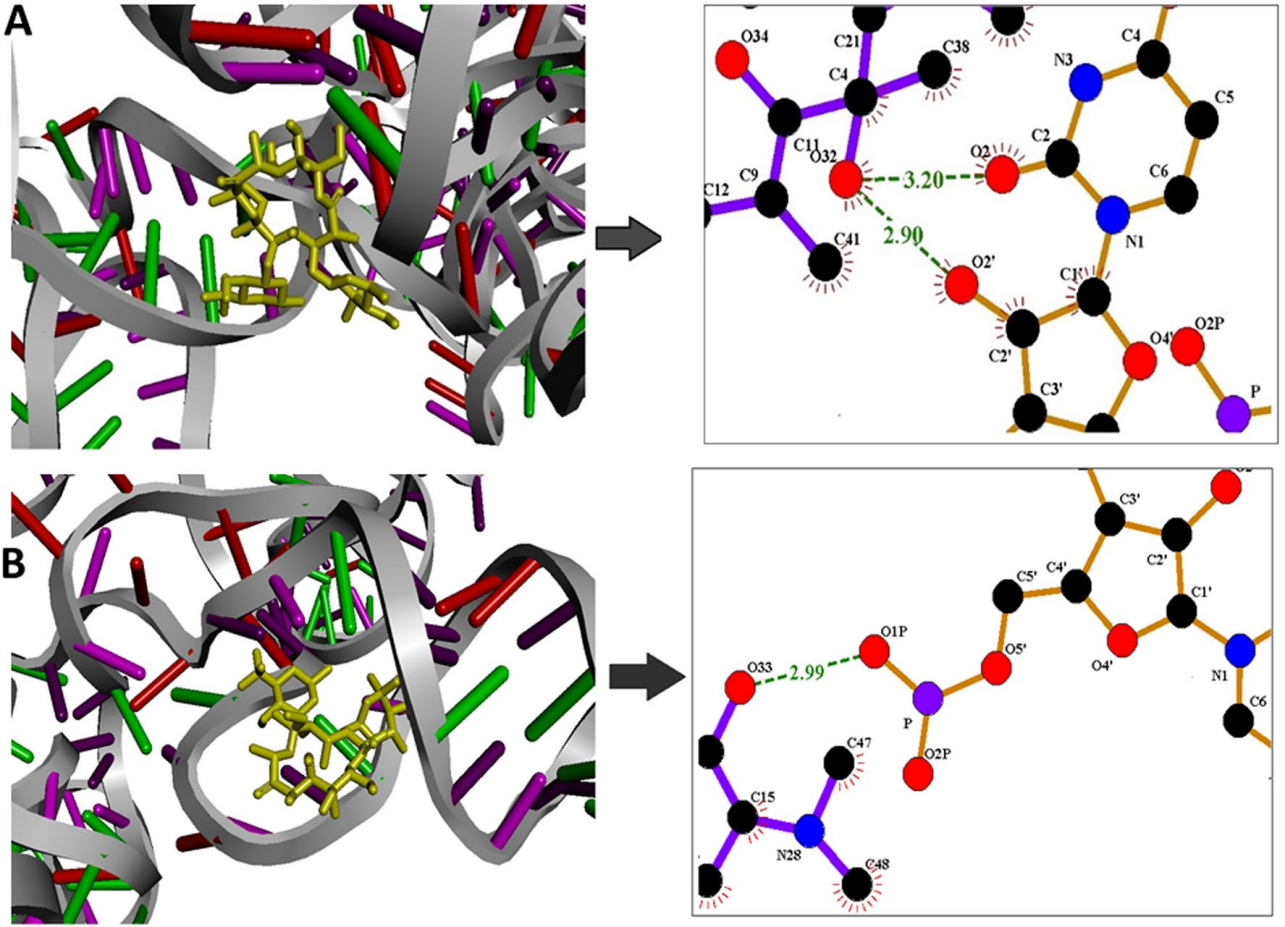
Interaction study of 23S rRNA of wild type (2143A) and mutant type (2143G) of *H. pylori* with clarithromycin: Green dotted lines represent hydrogen bond. **A** Interaction between 23S rRNA of wild type (2143A) *H. pylori* and clarithromycin showing more number of hydrogen bond imparting stronger binding. **B** Interaction between 23S rRNA of mutant type (2143G) type *H. pylori* and clarithromycin showing less number of hydrogen bond imparting weaker binding

We observed that 23S rRNA transcript containing 2143G corresponds to a more stable secondary structure. Therefore, it is expected that weaker interaction will be observed between the tertiary structure containing 2143G with CLR compared to the tertiary structure containing A2143. This is because 2143G is part of double stranded stem region where strand opening potential is also involved. On the other hand, A2143 is part of loop region; therefore, strand opening potential is not involved when it will react with CLR. Indeed, binding analysis of both wild (A2143) and mutant type (2143G) rRNA with CLR clearly showed that binding interaction of CLR is greater with the wild type (A2143) [Fig. 8(A)] than with the mutant type (2143G) (Fig. 8B) of 23S rRNA. It is also clear from these two figures [Fig. 8(A and B)] that a greater number of hydrogen bonds have been formed when 23S rRNA containing wild type (A2143) interacts with CLR compared to the interaction between 23S rRNA containing mutant type (2143G) with CLR; thereby providing stronger interaction between 23S rRNA containing wild type (A2143) with CLR than mutant type (2143G) with CLR. This result clearly demonstrates drug sensitivity is higher for the wild type (A2143) compared to mutant type (2143G).

## Discussion

The progressive rise in antibiotic resistance has become a big concern for treating the infectious diseases. The downfall in the *H. pylori* eradication rate can be attributed to its emerging resistant strains that correlate with its worldwide prevalence of infection and subsequent inflammation with neoplastic progression. CLR, one of the major macrolide, still serves as the most important antibiotic medicated against *H. pylori* treatment [22]. But the upsurge of CLR resistant *H. pylori* strains play a pivotal role in the decreasing success rates of *H. pylori* eradication therapy.

India is one of the largest consumers of antibiotics, and their use is not well regulated. Inapposite use of antibiotics directly trigger antibiotic resistance in India due to the lack of official monitoring of antibiotic usage. Largely unrestricted over-the-counter sales of most antibiotics complicate antibiotics availability, sales, and consumption in the country [50].

Following the onset of COVID-19 pandemic, azithromycin was earnestly used in the treatment protocol [51]. Due to huge demand of this antibiotic, situation reached to a stage when it was out of the stock for a certain period of time. Lately, wide enough shreds of evidences were gathered against the use of this extremely useful antibiotic azithromycin, for curing various clinical parameters in mild-to-moderate COVID-19 infections as indiscriminate use of the same would lead to serious antibiotic resistance problems [52]. During the COVID-19 pandemic the misuse and overuse of antibiotics specially macrolides being the subject of concern here, is two to three folds higher, as demonstrated in different studies [53, 54], adding fuel to the fire, posing the concern for developing more antibiotic-resistance in post COVID-19 era. Therefore, to preserve the effectiveness of existing antimicrobials, new measures of clinical guidelines along with the antibiotic stewardship principles are warranted on using macrolides and other antibiotics in developing countries including in India. Furthermore, these new implementations shall reach out well to the public and health practitioners to take over and balance the false information that all are receiving. This concern for increasing antibiotic-resistance after the COVID-19 pandemic will aggravate the treatment strategy for *H. pylori* infection.

The local prevalence of CLR resistance should be taken into consideration during the recommendation of first-line therapy for *H. pylori* infection and the subsequent treatment strategy should be sketched out by antimicrobial susceptibility testing [55]. Therefore, the methods for detecting antibiotic resistance are of great importance. It was observed that all the clarithromycin resistant *H. pylori* strains sequenced in our study had A2143G point mutation at 23S rRNA.

Our secondary structure analysis of the 23S rRNA peptidyl transferase gene containing A2143G polymorphism of wild type versus mutant revealed that this mutational change in conformation might lead to a change in drug binding probability. *In silico* analysis of the impact of mutation A2143G on 23S rRNA secondary structural change from loop into stem imparts higher stability of the 23S rRNA transcript containing 2143G. Respective accessibility changes in terms of unpaired probabilities and changes in base pairing probabilities suggest that rRNA secondary structure analysis is necessary to determine the effects of mutation. These analyses support our observation that stem formation is preferred because base pairing probabilities are higher. Thus, greater number of hydrogen bond found in wild type rRNA (A2143) compared to the mutant type rRNA (2143G) when interacting with the clarithromycin leading to the resistance. This “by chance” mutations poses no harm on bacterial fitness thus can be sustained for many generations. Moreover, this kind of mutation holistically affects all macrolides developing class-wide resistance [4]. Due to misinterpretations of these low proportion mutation by already existing methods present in *H. pylori* populations and further prescribing the wrong combination of antibiotics aggravate the situation through the selection of these resistant strains and promoting them to become the majority of bacterial populations.

This molecular assay addressing CLR resistance corroborated the result with phenotypic tests followed by sequencing, providing a significant correlation between the designed novel PCR assay and CLR resistance. Total CLR resistance found in this study was 8.04%. Moreover, the incidence of primary *H. pylori* resistance in the different populations across the world has been increasing progressively and woefully, varying between different geographical regions, as in Indian sub-populations. This percentage is believed to increase in the upcoming days due to widespread use of certain antibiotics (i.e., levofloxacin for urinary infections or CLR for respiratory infection). Besides this, the bacteria, *H. pylori* co-evolves with the highly genetically diverse Indian population and as a result, its response towards antimicrobials also keeps on changing. Most importantly, the variations in efficacy of the techniques used and interpretations; to detect the antimicrobial susceptibility patterns across different regions are highly prone in incorporating false positive results [30]. As an outcome, the resistant bacteria out-competes the sensitive ones and thrive in the gastric mucosa despite antibiotic application and leading to the disease procurement. All these issues lead to the unnecessary delay in curing of the bacterial infected individual. So this matter needs to get resolved with development of newer, rapid diagnostic approaches leading to precise identification of CLR resistant clinical *H. pylori* isolates before therapeutic implementation and further improvisations.

In this regards, molecular based assay proposed here not only gains advantages over traditionally used drug sensitivity tests but also acts as a useful tool for applying it in the clinical field and accurately identifying CLR resistance as well. Besides this, the assay may also be used as a validation tool for itself because the method developed here can also identify CLR-sensitive strains. In addition, this helps us to add knowledge to clinical antibacterial drug guidelines and reduce the economic burden on patients.

Maeda *et al.* described a semi-nested PCR to detect *H. pylori* infection from template DNA isolated from bacterial culture and gastric juices and then identified mutation at position 2143 and 2144 of 23S rRNA by restriction fragment length polymorphisms (RFLP) [37]. But in our cases, we proposed a novel PCR and concurrently nested-ASP-PCR to detect *H. pylori* infection along with the discrimination of CLR-resistant as well as sensitive isolates by using specific primers from culture as well as from biopsy specimens. It does not need any restriction enzyme for further clarification but only the allele-specific primers. Pan *et al.* proposed a primer-mismatched technique identifying CLR-resistant isolates from pure culture in China by exploiting mutational sequence divergence at nucleotide position 2143 of 23S rRNA [56]. However, we have developed a mismatch-amplification mutation PCR assay (MAMA PCR) to accurately discriminate CLR-resistant as well as sensitive strains based on single nucleotide polymorphisms (SNP) at nucleotide position 2143 in a rapid way. This method is suitable to use with pure culture and also with biopsy specimens utilizing the nested PCR principle. Furthermore, we also compared our PCR assay which deals with crude DNA prepared by the Phenol-chloroform extraction method and boiled lysate method with the one described by Pan *et al.* dealing with different methods of template preparation; phenol chloroform-isoamyl alcohol extraction and ethanol precipitation or with a QIAamp tissue kit (Qiagen Inc., Chats-worth, Calif). Additionally, in case of assay described by Pan *et al.* yielded non-specific resistant band along with specific ones whereas our assay produced only a specific band [56]. Zhang *et al.* reported the development of ARMS-PCR for quick detection of CLR resistance, which needs standardization with primers and fluorescent probes making it difficult right from the experimental design [57]. Moreover, testing costs limit its clinical application. Vazirzadeh *et al.* has described the development of Fluorescent In Situ Hybridization (FISH) in the Center of Iran for rapid detection of CLR resistance in *H. pylori* but this is a probe based method and requires standardization and is not easy to perform in different laboratories [58].

Our described novel PCR assay is appropriate to identify mutation at the 2143 nucleotide position of 23S rRNA in resistant isolates. The only limitation of our assay is that it can detect only A2143G mutation with 100% specificity and sensitivity but not any other SNPs on 23S rRNA (if at all present). In India, we have found so far only A2143G mutation in CLR resistant *H. pylori* strains. The result from other studies from different countries around the world reported 80 to 90% of the CLR resistant had A2143G mutations [24, 26, 28].

## Conclusion

The increasing prevalence of clarithromycin resistant *H. pylori* strains poses a great threat in eradication therapy. Our developed PCR assay exhibits a low cost and rapid detection method to detect point mutation that confers CLR resistance as well as CLR sensitive strains, providing rapid initiation of effective antibiotic treatment. The traditional culture method is highly time consuming and real time PCR, DNA sequencing and other techniques require experience and standardization and not suitable as a diagnostic tool. Therefore, this method can be used as a potential replacement for culture-based method. This would also aid to avoid blindly prescribing empirical treatment and in selection of rightly effective antimicrobial agents which reduces the probability of treatment failure and cost of treatment. Computational analysis depicted that resistant and sensitive strains have a differential binding affinity towards CLR which is corroborated by the variation of secondary structural stability due to point mutation A to G at the 2143 position of 23S rRNA of the sensitive and resistant strains respectively. The point mutation confers more stability in the secondary structure of the 23S rRNA of the resistant strain.

Finally, this study depicts a simple PCR based assay for rapid detection of point mutation directly from the biopsy samples leading to precise identification of CLR-resistant clinical *H. pylori* isolates before therapeutic implementation and further management.

## Data Availability

The sequences submitted in the Genbank are available with accession number MW080351 to MW080363. The original data and any other clarification for this work are available upon email request to the corresponding author.
